# Bullying victimization/perpetration and non-suicidal self-injury: A systematic review

**DOI:** 10.1192/j.eurpsy.2021.262

**Published:** 2021-08-13

**Authors:** G. Serafini, G. Canepa, A. Aguglia, A. Amerio, E. Flouri, M. Pompili, M. Amore

**Affiliations:** 1 Department Of Neuroscience, Rehabilitation, Ophthalmology, Genetics, Maternal And Child Health (dinogmi), University of Genoa, IRCCS Ospedale Policlinico San Martino, Genoa, Italy, Genoa, Italy; 2 Phd, UCL, London, United Kingdom; 3 Neurosciences, Mental Health And Sensory Organs, Sapienza University of Rome, Rome, Italy; 4 Department Of Neuroscience, Rehabilitation, Ophthalmology, Genetics, Maternal And Child Health (dinogmi), Departimento di Neuroscienze, Università di Genova, Genoa, Italy

**Keywords:** Bullying perpetration, Bullying victimization, non-suicidal self-injury, adolescents

## Abstract

**Introduction:**

Experience of bullying may be a significant risk factor for non-suicidal self-injury (NSSI).

**Objectives:**

This study had three aims: to systematically investigate the association between bullying and NSSI, to analyze the possible mechanisms underlying the two phenomena, and to evaluate any differences between bullying victimization and bullying perpetration with respect to NSSI.

**Methods:**

A systematic search about the association between bullying victimization and perpetration and NSSI was conducted using specific databases (PubMed, Scopus, Science Direct). The following keywords were used in all database searches: “bullying” AND “NSSI” OR “peer victimization” and NSSI.

**Results:**

The searches in PubMed, Scopus and Science Direct revealed a total of 88 articles about bullying or peer victimization and NSSI. However, only 29 met our inclusion criteria and were used for the present review. Overall, all studies examined victimization; 4 studies also evaluated the effects of perpetration and 1 included bully-victims. According to the main findings, both being a victim of bullying and perpetrating bullying may increase the risk of adverse psychological outcomes in terms of NSSI and suicidality in the short and the long run.

**Conclusions:**

To the best of our knowledge, this is the first review to systematically evaluate the relation between bullying victimization/perpetration and NSSI. The main results support a positive association. Future research should evaluate the possible role of specific mediators/moderators of the association between experience of bullying and NSSI.

**Disclosure:**

No significant relationships.

